# Mamao Pomace Extract Alleviates Hypertension and Oxidative Stress in Nitric Oxide Deficient Rats

**DOI:** 10.3390/nu7085275

**Published:** 2015-07-28

**Authors:** Upa Kukongviriyapan, Veerapol Kukongviriyapan, Patchareewan Pannangpetch, Wanida Donpunha, Jintana Sripui, Amporn Sae-Eaw, Orachorn Boonla

**Affiliations:** 1 Department of Physiology, Faculty of Medicine, Khon Kaen University, Khon Kaen 40002, Thailand; 2 Department of Pharmacology, Faculty of Medicine, Khon Kaen University, Khon Kaen 40002, Thailand; E-Mails: veerapol@kku.ac.th (V.K.); patc_pan@kku.ac.th (P.P.); 3 Department of Physical Therapy, Faculty of Associated Medical Science, Khon Kaen University, Khon Kaen 40002, Thailand; E-Mail: wanida_ams@yahoo.com; 4 Department of Food Technology, Faculty of Technology, Khon Kaen University, Khon Kaen 40002, Thailand; E-Mails: jinsri@kku.ac.th (J.S.); sampor@kku.ac.th (A.S.-E.); 5 Faculty of Allied Health Sciences, Burapha University, Chonburi 20131, Thailand; E-Mail: kukkaiorachorn@gmail.com

**Keywords:** *Antidesma thwaitesianum*, antioxidant, endothelial dysfunction, hypertension, l-NAME, Mamao pomace, nitric oxide, oxidative stress

## Abstract

Reactive oxygen species (ROS)-induced oxidative stress plays a major role in pathogenesis of hypertension. *Antidesma* *thwaitesianum* (local name: Mamao) is a tropical plant distributed in the tropical/subtropical areas of the world, including Thailand. Mamao pomace (MP), a by-product generated from Mamao fruits, contains large amounts of antioxidant polyphenolic compounds. The aim of this study was to investigate the antihypertensive and antioxidative effects of MP using hypertensive rats. For this purpose, male Sprague-Dawley rats were given *N*^ω^-nitro-l-arginine methyl ester (l-NAME), an inhibitor of endothelial nitric oxide synthase (eNOS), in drinking water (50 mg/kg) for three weeks. MP extract was orally administered daily at doses of 100 and 300 mg/kg. l-NAME administration induced marked increase in blood pressure, peripheral vascular resistance, and oxidative stress. MP treatment significantly prevented the increase in blood pressure, hindlimb blood flow and hindlimb vascular resistance of l-NAME treated hypertensive rats (*p* < 0.05). The antihypertensive effect of MP treatment was associated with suppression of superoxide production from carotid strips and also with an increase in eNOS protein expression and nitric oxide bioavailability. The present results provide evidence for the antihypertensive effect of MP and suggest that MP might be useful as a dietary supplement against hypertension.

## 1. Introduction

Epidemiological studies revealed that consumption of a healthy diet (e.g., fruits, vegetables and whole grains) decreases the risk of cardiovascular diseases [1]. Hypertension is one of the major risk factors of cardiovascular diseases. The incidence of hypertension is increasing worldwide. Previous reports forecast that by 2025, the rate of hypertension will increase by 26.4% to 60%, and will affect approximately 1.56 billion people worldwide [2]. Data from many countries in Southeast Asia, including Thailand, have also shown a trend of increasing hypertension [3].

Oxidative stress is involved in the pathogenesis of hypertension [4]. Vascular endothelial cells play a major role in arterial relaxation. Nitric oxide (NO), synthesized by and released from the vascular endothelium, is a potent vasodilator [5]. NO is rapidly degraded by the oxygen-derived free radical superoxide anion (O_2_^•−^). O_2_^•−^ is a major determinant of NO biosynthesis and bioavailability, and can modify endothelial function. Development of hypertension is closely associated with a decrease in NO bioavailability and an increase in oxidative stress [6]. Since oxidative stress is involved in hypertension, antioxidants have a beneficial effect on hypertension [7,8].

Dietary antioxidants such as polyphenols, carotenoids, tocopherols, tocotrienols, phytosterols, isoflavones, organosulphur compounds, *etc*., have protective effects against various diseases [9]. *Antidesma thwaitesianum Műell. Arg.* (Thai local name: Mamao Luang or Mamao) is an evergreen tree in Phyllanthaceae family. It is a tropical plant distributed in Africa, Southeast Asia, Australia and Oceanian islands [10]. The greatest diversity is in Southeast Asia. *Antidesma* fruits are acidic in taste but become sweet when fully ripe. Fruits of *A.* *thwaitesianum* are processed for juice, wine, jam, *etc.*, in food industries, producing by-products composed of seeds and marcs, so-called pomace. The pomace contains a number of polyphenols and anthocyanins [11]. Anthocyanins are one of six subgroups of flavonoids having strong antioxidant and anti-inflammatory activities [12,13]. Since oxidative stress is implicated in cardiovascular abnormalities [14,15], suppression of oxidant formation with dietary antioxidants can prevent or ameliorate the adverse cardiovascular events [16,17,18,19].

*Antidesma* *thwaitesianum* possesses strong antioxidant activity *in vitro* [11]. However, there is still scarce evidence supporting the beneficial effect of antioxidant activity of *A.* *thwaitesianum* on cardiovascular diseases. Using an l-NAME-induced hypertensive rat model, the present study investigated possible mechanisms by which antioxidants present in *A.* *thwaitesianum* pomace extract confer their beneficial antihypertensive effects.

## 2. Experimental Section

### 2.1. Plant Material and Sample Preparation

The fruits of *A.* *thwaitesianum* (Mamao Luang) were collected at Phuphan District, Sakon Nakhon, Thailand, from July to September 2013. The pomace of Mamao Luang (MP) was separated from the fruit, shade-dried and minced into coarse powder. The powder (250 g) was macerated with 95% ethanol (2.5 L) for 4 h, and then filtered. The ethanol was evaporated using a rotary evaporator. The ethanolic extract of MP was freeze-dried into powder and kept in an air-tight and light-protected container at −20 °C until use. Yield of the MP extract was 15.4% weight/weight (w/w), calculated based on the dry weight of fruits.

### 2.2. Antioxidant Assays

The antioxidant activity of MP was measured using diphenyl-2-picrylhydrazyl (DPPH) and ferric reducing antioxidant power (FRAP) assays as described previously [16]. In brief, MP dissolved in ethanol was mixed with DPPH (0.25 mM) and the absorbance at 515 nm was measured using ascorbic acid as a standard. The FRAP assay was performed using freshly prepared FRAP reagent comprised of 300 mM acetate buffer (pH 3.6), 10 mM 2,4,6-tripyridyl-s-triazine in 40 mM HCl; and 20 mM FeCl_2_ at a 10:1:1 ratio. The FRAP reagent was mixed with MP solution and the absorbance at 610 nm was measured. The FRAP value was calculated as reducing power of ascorbic acid. The total monomeric anthocyanin content was estimated using a pH differential method [20]. Absorbance was measured at 510 nm and 700 nm in KCl buffer (0.025 M, pH = 1.0) and sodium acetate buffer (0.4 M, pH 4.5). The differential absorbance at pH 1 and pH 4.5, A = (A_510_ − A_700_) pH1 − (A_510_ − A_700_) pH4.5, and the molar extinction coefficient of 26,900 M^−1^·cm^−1^ were used for calculation. Amount of monomeric anthocyanins was expressed as mg of cyanidin-3-glucoside/g MP.

### 2.3. *In Vivo* Assessment for the Effect of MP

Male Sprague-Dawley rats weighing 200–220 g were obtained from the National Laboratory Animal Center, Nakhon Pathom, Thailand. The rats were housed in the Heating, Ventilation and Air-Conditioning (HVAC) System with 12 h dark/light cycle in the Northeast Laboratory Animal Center, Khon Kaen University, Thailand, and were fed with a standard chow diet (Chareon Pokapan Co. Ltd., Samutprakarn, Thailand). The study protocols were reviewed and approved by the Animal Ethics Committee of Khon Kaen University (AEKKU28/2556). All surgical procedures were performed under standard anesthesia, and all efforts were made to minimize suffering.

After one week of acclimatization, rats were randomly divided into two main groups; the normal control group received tap water and the l-NAME-treated group received l-NAME (50 mg/kg/day) in drinking water for three weeks [17,21]. Rats from each group were given 100 mg/kg (MP100) or 300 mg/kg (MP300) of MP extract orally with deionized distilled water (DI) as vehicle once daily using a stomach tube. Accordingly, the experimental groups (*n* = 10/group) consisted of (1) normal control + DI; (2) normal control + MP100; (3) normal control + MP300; (4) l-NAME + DI; (5) l-NAME + MP100, and (6) l-NAME + MP300. The dose of MP was chosen based on our preliminary observation of the inhibitory effect of MP on l-NAME-induced hypertension in rats [22].

#### 2.3.1. Hemodynamic Measurements

The blood pressure of rats was monitored before and every week during the study period. Systolic blood pressure (SBP) of conscious rats was measured indirectly using the tail-cuff plethysmography (Blood pressure analyzer, model 29, IITC, Woodland Hills, CA, USA). On the last day of the experiment, rats were anesthetized with an intraperitoneal injection of pentobarbital sodium (60 mg/kg). A tracheotomy was performed for spontaneous breathing, and the left femoral artery was cannulated with a polyethylene catheter connected to a pressure transducer for continuous monitoring of blood pressure (BP), using the AcqKnowledge Data Acquisition and Analysis software (BIOPAC Systems Inc., Goleta, CA, USA). Baseline values of BP and heart rate were monitored for 10 min, and a second polyethylene catheter was inserted into the femoral vein to allow intravenous drug delivery.

For the continuous monitoring of hindlimb blood flow (HBF), an electromagnetic flow probe was placed around the abdominal aorta by laparotomy and connected to an electromagnetic flowmeter (Carolina Medical Electronics Inc., East Bend, NC, USA). After measurement of HBF, the hindlimb was cut and weighed. Hindlimb vascular resistance (HVR) was calculated from the mean arterial pressure divided by the hindlimb blood flow. HVR was expressed as 100 mg tissue units.

After obtaining stable baseline blood pressure data, vascular response to vasoactive agents was assessed by bolus infusion of the vasodilators; acetylcholine (ACh, 10 nmol/kg) and sodium nitroprusside (SNP, 3 nmol/kg), and vasoconstrictors; angiotensin II (Ang II, 0.3 nmol/kg) and phenylephrine (Phe, 0.1 mol/kg). The successive infusion of vasoactive agents was made after blood pressure returned to the baseline and stabilized for at least 5 min. At the end of the study, rats were sacrificed by overdose of anesthestic drug. Blood samples were withdrawn from the abdominal aorta and transferred into ethylenediaminetetraacetic acid (EDTA) tube to assess oxidative stress and antioxidant markers. The carotid arteries were rapidly excised and and O_2_^•−^ production in arterial tissue was measured using the lucigenin enhanced chemiluminescence method as described previously [17].

#### 2.3.2. Oxidative Stress Biomarkers

Lipid peroxidation, as measured by malondialdehyde (MDA) level, was estimated using thiobarbituric acid (TBA) as described previously [23]. In brief, 150 μL of each plasma sample was treated with 10% trichloroacetic acid, 5 mM EDTA, 8% sodium dodecyl sulphate (SDS), 0.5 μg/mL of dibutylhydroxytoluene. After incubation for 10 min, 500 μL of 0.6% TBA was added, and the mixture was boiled for 30 min. After cooling down to room temperature, the mixture was centrifuged at 10,000× *g* for 5 min. The absorbance of the supernatant was measured at 532 nm using a spectrophotometer. A standard curve was generated with appropriate concentrations of 1,1,3,3-tetraethoxypropane (0.3–10 μM).

Nitrite and nitrate, the end products of NO metabolism, were used as indices of nitric oxide synthase (NOS) activity. To assess NO metabolites in plasma samples, plasma protein was removed by ultrafiltration [23]. The nitrate in the sample was converted to nitrite by nitrate reductase and total nitrite was determined by reacting with Griess solution. The absorbance of the samples was measured at a wavelength of 540 nm.

Protein oxidation, assessed as the protein carbonyl, was measured as described previously [24]. Briefly, a plasma sample was reacted with 15 mM dinitrophenylhydrazine (DNPH) in 3.6 M HCl for 1 h in the dark. Protein was precipitated and dissolved in 6 M guanidine and the absorbance was read at 360 nm. The carbonyl content was determined from the absorbance of the DNPH-treated sample subtracted from that of the corresponding non-treated sample, using a molar extinction coefficient of 22,000 M^−1^·cm^−1^.

Vascular O_2_^•−^ production was determined using the lucigenin-enhanced chemiluminescence method as described previously [18]. The carotid arteries were rapidly dissected and cleaned of connective tissue. The arterial segments of 3–4 mm in length were incubated in Krebs-KCl buffer at 37 °C and lucigenin (100 M) was added. Photon counts were made on a luminometer (Turner Biosystems Inc., Sunnyvale, CA, USA). The arterial sections were dried for 24 h at 45 °C and weighed. The O_2_^•−^ formation was expressed as relative light unit counts per min per mg dry weight.

#### 2.3.3. Western Blot Analysis

Endothelial NOS (eNOS) in the aortic tissue was measured as described previously [18]. Briefly, the aortic vessel was removed rapidly and homogenized in cell lysis buffer on ice. Protein was separated on SDS-polyacrylamide gel electrophoresis and transferred to polyvinylidene difluoride membrane. The membrane was blocked with 5% skimmed milk in Tris buffered saline with 0.1% Tween-20 and incubated overnight with mouse monoclonal anti-eNOS (BD Biosciences, San Jose, CA, USA) and goat polyclonal anti-β-actin followed by horseradish peroxidase-conjugated antibodies against mouse and goat IgG, respectively. The membranes were incubated with enhanced chemiluminescence (ECL) substrate solution (Supersignal West Pico Chemiluminescent substrate). The densities of eNOS and β-actin bands were visualized and captured using an Image Quant 4000 (GE Healthcare, Pittsburgh, PA, USA). The eNOS protein expression level was normalized to β-actin from the same sample.

### 2.4. Statistical Analysis

Data were presented as mean ± standard error of the mean (SEM). Statistical differences were evaluated by one-way analysis of variance (ANOVA) followed by the Post-Hoc test with Student Newman-Keul’s test to determine the differences between groups. Statistical significance was defined as *p* < 0.05.

## 3. Results

### 3.1. Antioxidant Activity of MP

The ethanolic extract of MP showed a free radical scavenging activity in DPPH assay and antioxidant power in FRAP assay using ascorbic acid as a standard (Table 1). Crude extract of MP was 120 times less potent than ascorbic acid in DPPH assay. The total anthocyanin content was 4.87 mg/g as cyanidin-3-glucoside equivalence per gram of MP.

**Table 1:** Antioxidant activity and total anthocyanin content of Mamao pomace (MP).

Assay	Ascorbic Acid	MP
DPPH (IC50)	5.03 ± 0.21 (µg/mL) *	600 ± 10 (µg/mL)
FRAP		10.1 ± 0.9 (mg ascorbate/g MP)
		114.7 ± 10.2 (meq /kg MP)
Total anthocyanin content		4.87 ± 0.03 (mg cyanindin-3-glucoside/g MP)

*Values are mean ± standard error of the mean (SEM) (*n* = 5). DPPH, diphenyl-2-picrylhydrazyl; FRAP, ferric reducing antioxidant power.

### 3.2. Effect of MP on Hemodynamics in l-NAME-Induced Hypertension Rats

l-NAME treatment and MP extract administration did not elicit any apparent signs of systemic toxicity such as body weight changes or changes in the gross appearance of animals. The body weights of animals in all groups were not different and weight gain and gross appearance were normal (data not shown), suggesting that the doses of l-NAME and MP extract used in this study did not disturb the animals’ health.

**Figure 1:**
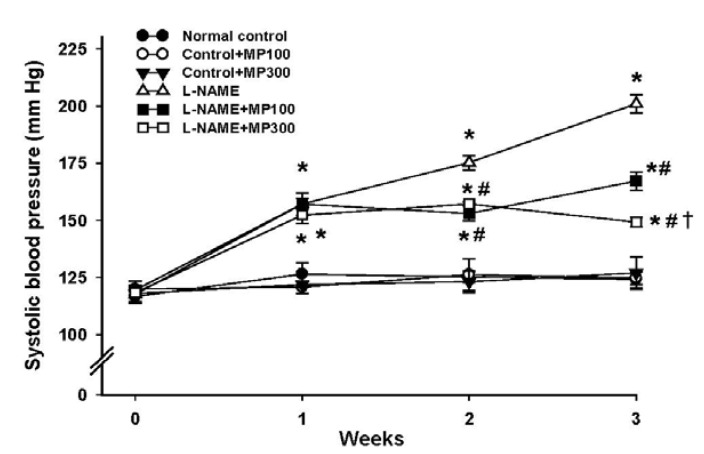
Effect of Mamao pomace extract (MP) on systolic blood pressure during *N*^ω^-nitro-l-arginine methyl ester (l-NAME)-induced hypertension. The blood pressure of normotensive and l-NAME hypertensive rats was measured for 3 weeks by tail-cuff plethysmography. MP was administered at doses of 100 (MP100) and 300 (MP300) mg/kg/day. Values are expressed as mean ± standard error of the mean (SEM) of 8–10 animals/group. * *p* < 0.05 *vs.* normal control group, **^#^** *p* < 0.05 *vs.* l-NAME control group, **†** *p* < 0.05 *vs.* l-NAME + MP100 group.

Administration of l-NAME via drinking water induced an increase of blood pressure in rats within the first week and the systolic blood pressure (SBP) increased progressively during the course of three-week treatment (Figure 1). Daily administration of MP prevented the rise in SBP and stabilized SBP on the second and third weeks (Figure 1).

Treatment with MP for three weeks prevented the increases in systolic, diastolic and mean blood pressures, while heart rate did not significantly change (Table 2). HBF was markedly reduced in the l-NAME group, but this reduction was prevented by MP. The HVR in l-NAME group was dramatically increased, but MP treatment normalized the resistance, particularly at the higher dose. Antihypertensive effect of MP extract after three weeks of treatment was dose-dependent. It should be noted that MP treatment did not affect the blood pressure, heart rate, HBF or HVR of normotensive control animals.

**Table 2:** Effect of MP on hemodynamics in N^ω^-nitro-l-arginine methyl ester (l-NAME)-induced hypertensive rats after three weeks.

Parameters	Normal Control			L-NAME		
	Vehicle	MP100 (mg/kg)	MP300 (mg/kg)	Vehicle	MP100 (mg/kg)	MP300 (mg/kg)
SBP (mmHg)	118 ± 3	128 ± 4	127 ± 4	192 ± 4 *	158 ± 4 *^,#^	141 ± 3 *^,#,^†
DBP (mmHg)	76 ± 3	77 ± 2	82 ± 3	134 ± 4 *	108 ± 3 *^,#^	90 ± 2 *^,#,^†
MAP (mmHg)	93 ± 3	96 ± 2	100 ± 5	156 ± 4 *	129 ± 2 *^,#^	112 ± 2 *^,#,^†
HR (beats/min)	368 ± 43	352 ± 21	383 ± 5	348 ± 26	386 ± 18	387 ± 13
HBF (mL/min/100 g tissue)	8.2 ± 0.5	8.3 ± 0.3	8.2 ± 0.5	4.2 ± 0.5 *	6.2 ± 0.4 *^,#^	7.3 ± 0.3 ^,#,^†
HVR (mmHg/mL/min/100 g tissue)	11.7 ± 0.9	11.9 ± 0.6	11.6 ± 0.8	44.3 ±5.7 *	21.0 ±1.9 *^,#^	12.4 ± 0.4 ^,#,^†

Values are mean ± SEM (*n* = 8–10/group); The data obtained at 3 weeks of treatment; * *p* < 0.05 *vs.* normal control group, ^#^ *p* < 0.05 *vs.* l-NAME control group, † *p* < 0.05 *vs.* l-NAME+ MP100 group; SBP, systolic blood pressure; DBP, diastolic blood pressure; MAP, mean arterial pressure; HR, heart rate; HBF, hindlimb blood flow; HVR, hindlimb vascular resistance. MP, Mamao pomace extract.

### 3.3. Effect of MP on Vascular Reactivity

**Figure 2:**
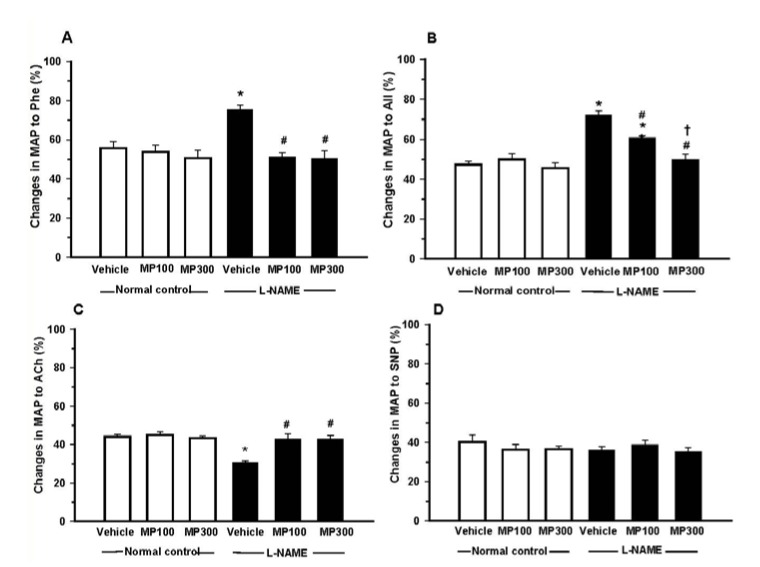
Effect of mamao pomace extract (MP) on vascular responsiveness in *N*^ω^-nitro-l-arginine methyl ester (l-NAME)-induced hypertensive rats. Changes in mean arterial blood pressure of rats in normal control and l-NAME-treated rats were measured. MP was administered at doses of 100 (MP100) and 300 (MP300) mg/kg/day. The vasoconstriction response to (**A**) phenylephrine (Phe) and (**B**) angiotensin II (AngII), and the vasodilation response to (**C**) acetylcholine (Ach) and (**D**) sodium nitroprusside (SNP) were measured. Each bar represents the mean ± standard error of the mean (SEM) (*n* = 8–10/group). * *p* < 0.05 *vs.* normal control group, **^#^** *p* < 0.05 *vs.* l-NAME control group, † *p* < 0.05 *vs*. l-NAME + MP100 group.

l-NAME-treatment induced an impairment of vascular responses to vasoactive agents in rats. The blood pressure response to vasoconstrictors, phenylephrine (Phe) and angiotensin II (AngII) was significantly augmented in l-NAME-treated rats (Figure 2A,B). MP-treatment at both low and high doses significantly reduced the pressor responses to Phe and AngII to the normal levels. Like-wise, the response to endothelial-dependent vasodilator, ACh, was significantly blunted in l-NAME-treated rats, and MP-treatment could normalize the response to the normal level (Figure 2C). However, the vasodilation response to SNP, an endothelial-independent vasodilator, was comparable between normal control and l-NAME-treated rats and was not affected by MP-treatment (Figure 2D).

### 3.4. Effect of MP on Oxidative Stress in L-NAME-Treated Rats

**Figure 3:**
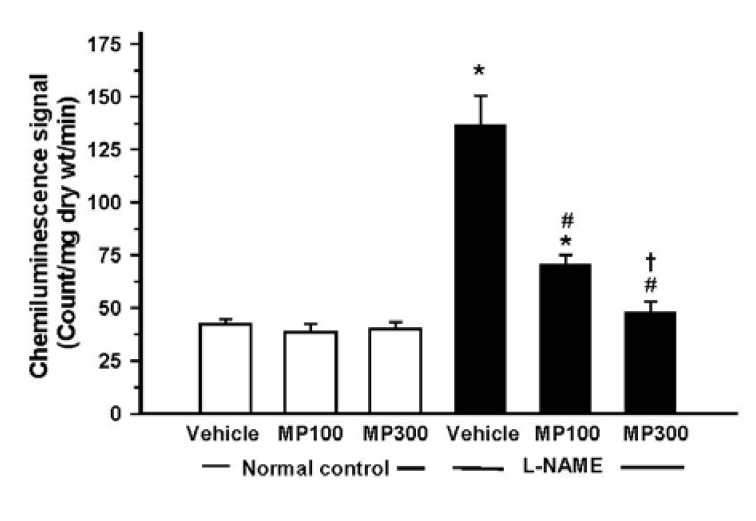
Protective effect of mamao pomace extract (MP) on superoxide formation in the carotid arteries of rats. The superoxide formation from carotid arteries of rats in normal control and *N*^ω^-nitro-l-arginine methyl ester (l-NAME)-treated rats was determined by chemiluminescence method. MP was administered at doses of 100 (MP100) and 300 (MP300) mg/kg/day. Each bar represents the mean ± standard error of the mean (SEM) (*n* = 8–10/group). * *p* < 0.05 *vs.* normal control group, ^#^ *p* < 0.05 *vs.* l-NAME control group, † *p* < 0.05 *vs.* l-NAME + MP100 group.

**Figure 4:**
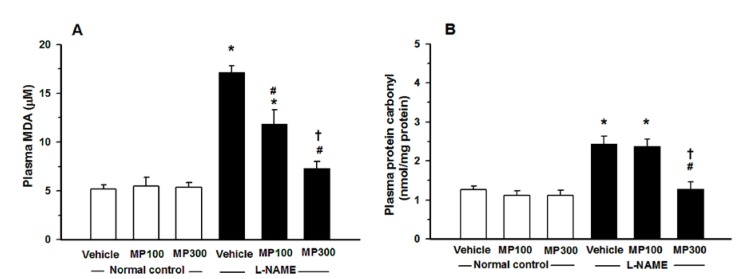
Protective effect of mamao pomace extract (MP) on oxidative stress. Plasma malondialdehyde (MDA) (**A**) and protein carbonyl (**B**) in normal control and *N*^ω^-nitro-l-arginine methyl ester (l-NAME)-treated rats were analyzed. MP was administered at doses of 100 (MP100) and 300 (MP300) mg/kg/day. Each bar represents the mean ± standard error of the mean (SEM) *(n* = 8–10/group). * *p* < 0.05 *vs.* normal control group, **^#^** *p* < 0.05 *vs*. l-NAME control group, † *p* < 0.05 *vs.* l-NAME+ MP100 group.

l-NAME treatment caused oxidative stress in rats, manifested as an increase in O_2_^•−^ formation, lipid peroxidation and protein oxidation. The l-NAME-treated rats showed a remarkable increase in the release of O_2_^•−^ from carotid arteries, and this was inhibited by MP treatment (Figure 3). Plasma MDA and plasma protein carbonyl were significantly increased in l-NAME rats and MP treatment prevented these increases (Figure 4A,B).

### 3.5. Effect of MP on l-NAME-Induced Suppression of Nitric Oxide Formation

**Figure 5:**
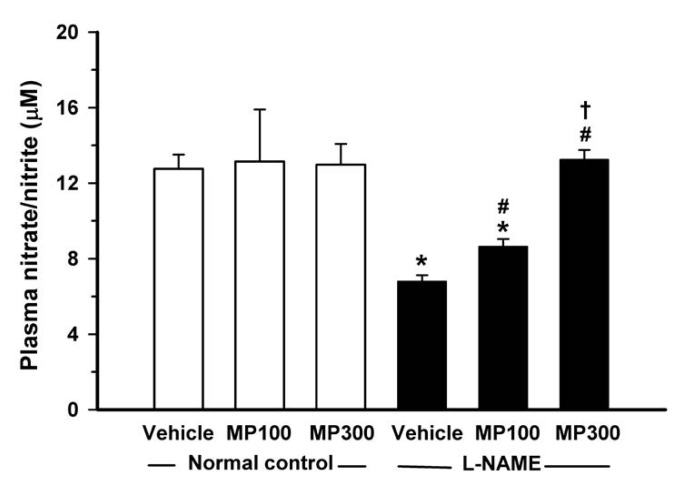
Effect of Mamao pomace extract (MP) on plasma nitrate/nitrite levels. Plasma nitrate/nitrite in normal control and *N*^ω^-nitro-l-arginine methyl ester (l-NAME)-treated rats were compared using an enzymatic method. MP was administered at doses of 100 (MP100) and 300 (MP300) mg/kg/day. Each bar represents the mean ± standard error of the mean (SEM) (*n* = 8–10/group). * *p* < 0.05 *vs.* normal control group, **^#^** *p* < 0.05 *vs.* l-NAME control group, † *p* < 0.05 *vs*. l-NAME + MP100 group.

NO plays important roles in several vascular functions. Levels of plasma nitrate/nitrite, metabolites of NO, were decreased by about 50% in l-NAME-treated rats compared with that in normal controls (Figure 5). MP-treatment restored the plasma nitrate/nitrite to levels similar to those in normal controls.

### 3.6. Effect of MP on eNOS Protein Expression

**Figure 6:**
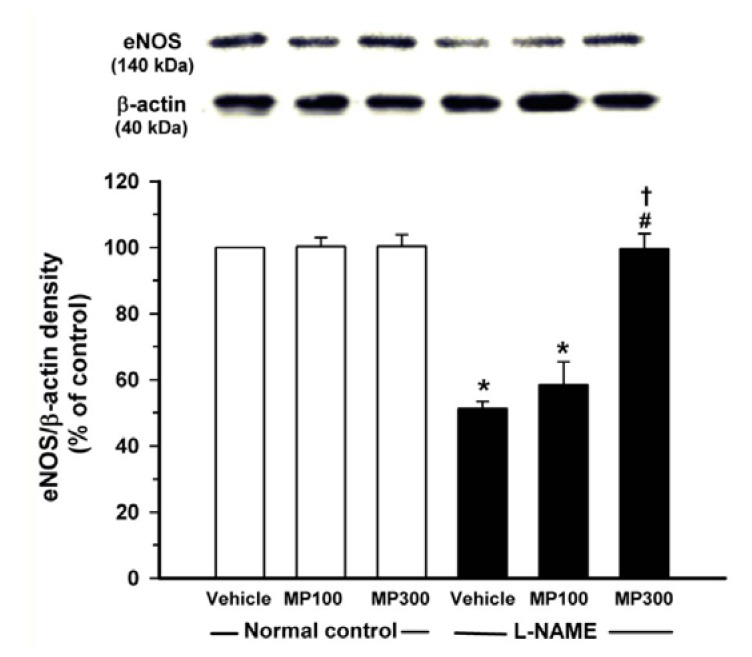
Protective effect of mamao pomace extract (MP) on endothelial nitric oxide synthase (eNOS) protein expression. Total protein lysates from normal control and *N*^ω^-nitro-l-arginine methyl ester (l-NAME)-treated rats were analyzed for eNOS by Western blotting. MP was administered at doses of 100 (MP100) and 300 (MP300) mg/kg/day. Each bar represents the mean ± standard error of the mean (SEM) (*n* = 5/group). * *p* < 0.05 *vs.* normal control group, **^#^** *p* < 0.05 *vs*. l-NAME control group, † *p* < 0.05 *vs.* l-NAME + MP100 group.

The eNOS protein expression in the aortic tissues was analyzed using Western blotting. l-NAME treatment caused the down-regulation of aortic eNOS protein expression. MP-treatment restored the eNOS expression level in a dose-dependent manner (Figure 6).

## 4. Discussion

*Antidesma* *thwaitesianum* fruits are used in several food and health products in many countries. Pomace from the fruit is a by-product containing a number of active phytochemicals. The ethanolic extract of the plant consists of high content of anthocyanins and possesses a moderate radical scavenging and reductive activity. MP showed potent antioxidant activity and prevents oxidative stress in hypertensive animals. MP also possesses antihypertensive effect and prevents vascular dysfunction in l-NAME-treated rats. The protective effect on vascular dysfunction was associated with the normalization of O_2_^•−^ and NO production and eNOS protein expression.

The antiradical activity and reductive activity of MP, which were assessed by DPPH assay and FRAP assay, respectively, were relatively moderate when compared with those of other colored fruits such as blueberry, blackberry, strawberry and pomegranate [25]. However, MP contains extraordinary high amount of anthocyanins compared with the aforementioned fruits or other vegetables (4.9 mg *vs.* 1–2 mg cyanidine-3-glucoside/g, respectively) [25,26]. Anthocyanins are known to have a protective effect against oxidative stress and cardiovascular dysfunction, particularly in the endothelium, by contributing to vascular homeostasis [27,28]. The presence of anthocyanins, catechin and small molecules of phenolic compounds in MP was previously reported [11]. Anthocyanins can modulate oxidative stress *in vitro* and *in vivo* via up-regulation of antioxidant genes through the Nuclear factor E2-related factor 2/antioxidant response element (Nrf2-ARE) signaling pathway [12,29]. Anthocyanins also have anti-inflammatory activity via inhibition of the expression and secretion of proinflammatory cytokines through the inhibition of nuclear translocation of nuclear factor-κB (NF-κB) [13]. Apart from the direct chemical radical scavenging activity, anthocyanins and many other phytochemicals exert an antioxidant effect indirectly by increasing the expression of cellular antioxidant and cytoprotective genes, resulting in cytoprotective and anti-inflammatory effects [30,31]. This may explain, at least in part, the discrepancy between radical scavenging activity of MP expressed *in vitro* and antioxidant and vascular protection activity *in vivo*.

l-NAME-induced hypertension in rats involves the inhibition of NOS enzymes, causing suppression of NO formation and aortic cyclic guanosine monophosphate (cGMP) content, which in turn causes elevation of arterial blood pressure [32]. Endogenous NO plays a pivotal role in controlling vascular tone via induction of vasodilation. Moreover, NO also has important antioxidant, anti-inflammation, hemostasis and immunological functions [14]. Inhibition of NOS is known to cause the activation of vascular angiotensin converting enzyme, which eventually stimulates nicotinamide adenine dinucleotide phosphate (NADPH) oxidases and leads to an increase in ROS formation [33,34]. The diminished NO bioavailability and exaggerated ROS formation are associated with vascular endothelial dysfunction and hypertension, which is evident by diminution of ACh-induced vasodilation [17,18]. In this study, MP treatment clearly inhibited the development of hypertension. Since MP did not alter the blood pressure of normotensive control rats, MP itself has no direct hypotensive effect, but rather acts as an antihypertensive agent. This indicates that MP acts to maintain normal level of blood pressure and can reduce hypertension caused by oxidative stress. Most antioxidant nutrients have anti-hypertensive activity [35]. Consistent with the antihypertensive effect, MP treatment improved the vascular response to an endothelial-dependent vasodilator, ACh, to the normal control level, whereas the vascular response to SNP, a vascular endothelial-independent vasodilator, was not affected. Thus, the effect of MP on hypertension is associated with the improvement of endothelial dysfunction [17,18].

It should be noted that MP restored not only the vasodilation response to ACh, but also normalized the exaggerated vasoconstrictor responses to Phe and Ang II. The enhanced vasoconstriction response in l-NAME treated rats may be related to O_2_^•−^ formation [36]. The antioxidant effect of MP as shown by suppression of O_2_^•−^ formation in the vascular tissues may explain the modulatory effect of MP on the exaggerated vasoconstriction of Phe and Ang II. The restoration of vascular endothelial-dependent vasodilation and vasoconstriction tone by MP is consistent with the observation that MP normalizes vascular resistance, as measured by HVR. In the present study, eNOS expression and plasma nitrate/nitrite levels were restored to normal levels after MP-treatment. Blood pressure was also significantly reduced but not completely normalized after MP-treatment. These results suggest that, although the major antihypertensive effect of MP may be mediated via the upregulation of eNOS and increase in NO bioavailability, other possible mechanisms of MP such as inhibition of sympathetic over activity and inactivation of renin-angiotensin system by MP should be considered, because those two pathways can be activated by l-NAME to induce hypertension [37].

In this study, we assessed the oxidative stress by measuring the levels of MDA, protein carbonyl and superoxide in all experimental groups. Although the specificity of spectrophotometric methods to measure MDA and protein carbonyl assays is controversial, these assay methods are still most widely used to determine lipid peroxidation and protein oxidation. In this study, in addition to MDA and protein carbonyl, we used superoxide level as an additional marker to assess oxidative stress. The results show that the level was correlated well with the MDA and protein carbonyl levels. Oxidative stress in the l-NAME-treated rats was evident by the increase of various oxidative biomarkers, *i.e.*, O_2_^•−^, MDA and protein carbonyl. In this study, all oxidative biomarkers were improved after MP treatment. The amelioration of oxidative stress condition is associated with the improvement of vascular endothelial function, whereas NO deficiency and O_2_^•−^ may play critical roles in pathogenesis.

NO, as measured by its metabolites levels, was significantly decreased in l-NAME-treated rats, and MP treatment could restore NO to a normal level. As NO bioavailability could be decreased by its interaction with O_2_^•−^ resulting in peroxynitrite (ONOO^−^) formation, the NO deficiency is accentuated by O_2_^•−^ formation. ONOO^-^ has an additional detrimental effect on vascular function via its strong oxidant activity [15]. The antioxidant effect of MP may partly explain the restoration of NO availability by suppression of O_2_^•−^ and ONOO^−^ formation. In this study, eNOS protein expression, detected by Western bloting, was diminished in l-NAME-treated rats, as was shown in previous reports [17,18,38], suggesting the down-regulation of eNOS expression under oxidative stress. Generally, NO is an effective antioxidant as it is freely diffusible into cellular compartment. NO is a chain-breaking antioxidant in lipid peroxidation and inhibits ferrous-driven Fenton reaction. Decreased NO production/release leads to generation of ROS and alteration of the redox status. Since MP is an ROS scavenger, MP might inhibit the down-stream oxidants and increase NO bioavailability, as evidenced by the increase of eNOS, the key enzyme of vascular homeostasis. This may thereby prevent endothelial dysfunction, improve vascular reactivity and reduce hypertension.

## 5. Conclusions

The present results demonstrate that the MP from *Antidesma thwaitesianum* possesses strong antioxidant activity. This antioxidant activity may be related to its high anthocyanin content. The pharmacological effects of MP in l-NAME-induced hypertensive rats included antihypertensive effect and the preservation of vascular function. The effects of MP were associated with suppression of O_2_^•−^ formation, prevention of lipid peroxidation and protein oxidation, and the restoration of eNOS protein expression and NO bioavailability. The overall findings of this study suggest the potential use of MP as the dietary supplement for prevention and treatment of hypertension.
